# Kawasaki disease: two case reports from the Aga Khan Hospital, Dar es Salaam-Tanzania

**DOI:** 10.1186/s12887-018-1306-5

**Published:** 2018-10-23

**Authors:** Mariam Noorani, Nuruddin Lakhani

**Affiliations:** Aga Khan Hospital, Dar es Salaam, Tanzania

**Keywords:** Kawasaki, Vasculitis, Coronary aneurysms, Tanzania, Intravenous immunoglobulin, Rash, Fever

## Abstract

**Background:**

Kawasaki disease is a common childhood vasculitis which may result in cardiovascular morbidity if not adequately treated. Its epidemiology in the African region is not well described. Its features may mimic other childhood infections and hemoglobinopathies and it is rarely diagnosed in the East African region. These are the first reports of this disease from Tanzania.

**Case presentation:**

We present two cases of complete Kawasaki disease seen over a 2 year period and diagnosed as per the criteria defined by the American Heart Association. One child was and infant and the other a 3 year old. Both of them presented with a prolonged fever and mucocutaneous findings. None of the children developed coronary artery aneurysms. One was treated with aspirin alone and the other with both aspirin and intravenous immunoglobulin. Both children had complete recovery and did not have any cardiovascular sequelae.

**Conclusion:**

Kawasaki disease may be more common in the East African region than previously thought. It should be considered as a differential diagnosis in children who present with a prolonged fever of greater than 5 days and mucocutaneous findings. More awareness about this condition, its epidemiology, diagnosis and management are required in order to prevent the cardiovascular morbidity associated with it.

## Background

Kawasaki disease is one of the most common vasculitides in childhood. It occurs predominantly in infants and young children and has long term cardiovascular morbidity due to coronary artery lesions if not adequately treated [1]. It is the leading cause of acquired heart disease in children in developed countries. The highest incidence is in Asia with almost 1 in a 100 children in Japan having the disease by age 5 [2]. In Africa, two cases were reported in South Africa in Caucasian children in 1980 but the first case in an African child was reported in Ivory Coast in 1981. Despite of sporadic cases being reported across many countries in Africa, mainly North Africa and the West African region, the epidemiological data for KD is limited for African countries [3–5].

The etiology of the disease remains unknown and several hypothesis exist in trying to explain the cause. Epidemiologic data suggest that an infectious agent may be causing the disease in genetically susceptible individuals [6]. The classic presentation of the disease occurs in children below 5 years. This suggests that there is an environmental trigger to which children mount an immune response after which the disease no longer manifests [7]. Postulated infectious agents include variants of normal flora which are induced by environmental factors such as improved hygiene [8]. Pathogenetic mechanisms postulated to result in endothelial injury include that of a protein homeostasis system in which immune cells target pathogenic proteins bound to endothelial cells resulting in host cell injury [9].

The initial symptoms are of a febrile illness lasting longer than 5 days with other mucocutaneous features including a rash, conjuctivitis and adenopathy. Diagnostic criteria exist to make a diagnosis of both complete and incomplete forms of disease [10]. If left untreated, the symptoms resolve in about 10 days. However, the coronary artery lesions may lead to long term cardiovascular complications including myocardial infarction, heart failure and arrhythmias. Treatment in the acute phase reduces the incidence of coronary artery lesions from 25 to 30% to 3–5% [11]. Currently, the recommended treatment is intravenous immunoglobulin in addition to acetylsalicylic acid at high doses [10].

In sub-saharan Africa, children suffer from many tropical and infectious diseases that present with symptoms similar to those of kawasaki disease such as fever, irritability and a rash. Common conditions include malaria, typhoid fever, meningitis and viral exanthems such as measles and roseola infantum. Other conditions that may mimic this disease include sickle cell dactylitis and infection with group A beta hemolytic streptococcus. A high index of suspicion is hence required to identify cases of kawasaki disease and institute early treatment.

We report here 2 cases of kawasaki disease seen at our institution that met diagnostic criteria defined by the American Heart Association. These are the first reports in published literature from the East African region.

### Case presentations

#### Case 1

A.M was a 3 year old female child of African ethnicity who presented in July 2012 with a 8 day history of high grade fever and a 1 day history of swelling of the hands and feet. (Table 1 – timeline of case 1). She had received oral antibiotics, anti malarials and antihistamines with no improvement in symptoms. Her past medical history had been uneventful and her vaccinations were uptodate.

**Table 1:** Timeline of Case 1

Dates	Relevant past medical history and interventions		
	Previously well child with no chronic disease, growth and development appropriate for age, vaccinations complete as per schedule		
Date	Summary from initial and follow up visits	Diagnostic testing	Interventions
July 2012 – Day 1	8 days of fever 1 day of swollen hands and feet Received antibiotics, antimalarials, antihistamines On examination: dry lips, non-pitting edema, cervical nodes Diagnosis – probable sickle cell anaemia	WBC – 36,000/μl, platelets – 380,000/μl, hemoglobin-9.1 g/dl Blood cultures – negative (results on day 5) Sickling test – negative Peripheral smear – normal Reticulocyte count - normal	IV ceftriaxone
Day 3	Persistent fever. Maculopapular rash on chest. Diagnosis – probable Kawasaki meeting 3 of 5 criteria	Echocardiogram – normal coronary arteries Repeat blood count: elevated platelets – 644,000/μl	High dose aspirin –80 mg/kg/day
Day 5	Persistent fever		Flew to Nairobi for IVIG treatment
Day 7	Fever resolved	Repeat Echo normal	Discharged on low dose aspirin – 5 mg/kg/day
September 2012	Follow up visit – no signs and symptoms	Echo – normal WBC and platelets – normal	Aspirin stopped

On examination, she was alert, had dry, red lips and non pitting edema on her hands and feet. She had cervical nodes measuring about 0.5 cm. Her cardiovascular exam was normal. She was admitted for further work up for the cause of her fever.

Her results showed an elevated WBC count of 36,000/μl, Hb of 9.1 g/dl and platelets of 380,000/μl. Her CRP was 173 mg/l. Malaria antigen and slide were both negative. Her urinalysis was normal. She was started empirically on ceftriaxone for presumed bacteremia and blood and urine cultures were sent. A differential diagnosis of sickle cell anaemia with dactylitis was also made and a peripheral smear, reticulocyte count and sickling test were requested. The reticulocyte count was low with a percentage of 0.38. Sickling test was negative and the peripheral smear was normal.

She continued to have fever spikes despite the antibiotics and then developed a maculopapular hyperemic rash on her chest. A diagnosis of incomplete kawasaki disease was now made which met 3 out of the 5 required criteria. An echocardiogram was done which showed normal coronary arteries. High dose aspirin was started at 80 mg/kg/day. IVIG was not available at the institution at that time. A repeat complete blood count showed some improvement in white blood cells (28000/μl) but elevated platelets of 644000/μl. The blood and urine culture were both reported as negative after 48 h.

The child was then flown out to Nairobi, Kenya for IVIG treatment which she received uneventfully. She was discharged on low dose aspirin and subsequent echocardiograms remained normal.

#### Case 2

W.I was an 8 month old infant of African ethnicity who presented in August 2013 with a 6 day history of a high grade fever with temperatures upto 39 degrees celsius. (Table 2 – Timeline of case 2). This was associated with redness of the eyes, lips and mouth. He had been treated at various health facilities with antimalarials and 2 antibiotics: cefalexin and coamoxiclav. He had also received paracetamol, ibuprofen and diclofenac injections to control the fever. His past medical history was uneventful and his vaccines were uptodate. He had attained milestones appropriately.

**Table 2:** Timeline of Case 2

Dates	Relevant past medical history and interventions		
	Previously well child with no chronic disease, growth and development appropriate for age, vaccinations complete as per schedule		
Date	Summary from initial and follow up visits	Diagnostic testing (including dates)	Interventions
August 2013 – day 1	Fever for 6 days and redness of eyes, lips and mouth. Treated with antimalarials, antibiotics with no relief On examination: dry cracked lips, conjuctival injection, rash at BCG scar site and on the neck Diagnosis: incomplete kawasaki	Platelets: normal – 190,000/μl Malaria negative Echo: normal coronary arteries	High dose aspirin – 90 mg/kg/day
Day 3	Fever resolved Swelling of hands and feet Diagnosis: complete kawasaki	Platelets elevated – 743,000/μl CRP elevated: 153 mg/l	Aspirin reduced to 5 mg/kg/day
Day 7	Swelling subsided Skin exfoliation	Repeat platelet count: 605,000/μl CRP: reduced to 60 mg/l	Aspirin continued
Day 30	Follow up visit – no signs and symptoms	Repeat echo: normal CRP - < 5 mg/l Platelets: normal range	Aspiring stopped

On examination, the child was irritable and difficult to console. He was febrile with a temperature of 38.5 ^0^ C and was not pale. He had dry, cracked, hyperemic lips and conjuctival injection bilaterally. Cervical nodes were not palpable. He had a papular rash on the neck and at the site of the BCG scar. The rest of his physical exam was normal.

A presumptive diagnosis of incomplete kawasaki disease was made meeting 3 of the five criteria required in addition to the fever. A complete blood count showed mild anaemia with normal platelet count. (WBC: 9.07 *10^3^/μl, Hb: 9.3 g/dl, platelets:190,000/μl). Malaria antigen and blood slide were both negative. He was started on high dose aspirin at 90 mg/kg/day as IV IG was not available in our institution at that time. An echocardiogram was done which showed normal coronary arteries and normal cardiac function.

His fever resolved after 72 h and he developed swelling of the hands and feet which now confirmed the diagnosis of complete kawasaki disease. The aspirin dose was reduced to 5 mg/kg/day after the fever subsided. The platelet count was repeated and it showed elevated platelets of 742,000/μl. The CRP was also high: 153 mg/l.

On follow up after a week, the limb swelling had reduced and he had skin exfoliation. His CRP had reduced to 60 mg/l and the platelets were 605,000/μl.

A follow up echo done after 1 month was normal and the aspirin was stopped after the hematological parameters had normalised. He has subsequently remained well with no cardiovascular sequelae.

## Discussion and conclusions

Both cases described above presented to us after a prolonged duration of fever and failure to respond to antimalarials and antibiotics. This is a common presentation also described in case reports from Ghana [3].

The first case was 3 year old child while the second was an infant. This is in keeping with epidemiologic studies from Algeria and Japan which have shown that more than 90% of children are less than 5 years of age [2, 5].

The children had the classical mucocutaneous features that are key to making the diagnosis. The first child had limb swelling, a skin rash and oral mucosal changes. The limb swelling was initialy thought to be sickling dactylitis which is a common condition seen in our environment and is one of the presenting signs of sickle cell disease [12].

The second child had non purulent conjuctivitis, swelling of limbs and the skin rash with involvement of the BCG scar which has been described previously [13]. Hematologic parameters are not diagnostic of kawasaki disease, however they can assist to confirm or support the diagnosis. Both our children were admitted with normal platelet counts which subsequently became elevated. This is a common finding in the second week of illness and is used as an adjunct in making diagnosis of incomplete kawasaki disease [10]. The first child had an elevated WBC count leading to a diagnosis of bacteremia and initiation of parenteral antibiotics. However, the blood culture eventually ruled out a bacteremia. The CRP was raised for both the cases as expected and eventually reduced after initiation of aspirin.

The first case was treated with both aspirin and IVIG while the second received only aspirin. The role of aspirin remains uncertain in the current era of use of IVIG, however it still remains as part of the standard treatment protocols. The current treatment: IVIG is an expensive drug that is not readily available or affordable to most families in our population. A single vial of 5 g costs as much as 1 million tanzanian shillings which is about 450 US dollars. This hampers effective treatment which is essential to reduce the cardiovascular sequelae.

Other treatment options that could be considered are use of corticosteroids. A cochrane review of 7 trials showed benefits of reducing cardiac complications by using steroids as adjunct therapy to IVIG early in the course of illness [14]. Use of steroids alone without IVIG has not been studied. However, in low resource settings where IVIG is not available, steroids could be used to reduce the cardiovascular morbidity [15]. Other agents such as infliximab have shown promise as adjuncts to IVIG but are also not widely available in sub – saharan Africa [16].

The echocardiograms for both children were normal at baseline and on subsequent follow ups. This was reassuring since the incidence of coronary abnormalities is as high as 25–30% in patients who are not treated with IVIG [11]. Echo is a vital modality to follow up the cardiac sequelae in chidlren with kawasaki disease. However, it is not widely available in most resource limited settings and requires specialised training to operate. In Tanzania, it is only available at a few referral hospitals in the cities. This is yet another challenge in providing long-term care to children with kawasaki disease.

Kawasaki disease has not commonly been described in sub-saharan Africa. However, these 2 cases were seen over a 2 year period at a single tertiary institution. This raises concern about how many missed cases there may be which are not diagnosed or managed effectively since the fever and mucocutaneous changes eventually resolve within 3 weeks even without specific treatment. The subsequent devastating cardiovascular morbidity of this condition in children with missed diagnosis remains unknown.

The pattern of disease in the setting of tropical infections like malaria also remains unknown. Kawasaki like syndromes have been described in adults with HIV infection especially with severe immunosupression [17]. Similar illness in children infected with HIV has not been described but may also occur.

We recommend that a diagnosis of kawasaki disease be entertained for any febrile illness lasting longer than 5 days and presenting with mucocutaneous findings. Any child presenting with a prolonged fever should promptly be referred to and evaluated at a tertiary centre. Doctors and other care providers should be made familiar with this condition in order to diagnose, treat and prevent the cardiovascular morbidity and mortality.

Cases that are seen at other institutions should be published in literature so that the spectrum of the disease, its epidemiologic characteristics and its outcomes in sub-saharan Africa can be described. Equally important is that IVIG be made available at a subsidised and more affordable cost in order to benefit patients with kawasaki disease and other autoimmune conditions where it is indicated.

## Abbreviations

CBC: Complete blood count
IVIG: Intravenous immunoglobulin
WBC: White blood cells
